# Reduced rainfall and resistant varieties mediate a critical transition in the coffee rust disease

**DOI:** 10.1038/s41598-022-05362-0

**Published:** 2022-01-28

**Authors:** Kevin Li, Zachary Hajian-Forooshani, Chenyang Su, Ivette Perfecto, John Vandermeer

**Affiliations:** 1 School for Environment and Sustainability, University of Michigan, 440 Church St., Ann Arbor, MI 48109 USA; 2 Department of Ecology and Evolutionary Biology, University of Michigan, Ann Arbor, MI 48109 USA

**Keywords:** Agroecology, Climate-change ecology, Ecological epidemiology, Theoretical ecology

## Abstract

Critical transitions, sudden responses to slow changes in environmental drivers, are inherent in many dynamic processes, prompting a search for early warning signals. We apply this framework to understanding the coffee rust disease, which experienced an unprecedented outbreak in Mesoamerica in 2012–2013, likely a critical transition. Based on monthly infection data from 128 study quadrats in a 45-ha plot in southern Mexico from 2014 to 2020, we find that the persistent seasonal epidemic following the initial outbreak collapses in an evident subsequent critical transition. Characteristic signals of “critical slowing down” precede this collapse and are correlated with reduced rainfall, as expected from climate change, and planting of rust-resistant varieties, an ongoing management intervention. Recoveries from catastrophes may themselves be experienced as a critical transition and managers should consider the larger dynamical landscape for the possibility of subsequent transitions. Early warning signals could therefore be useful when evaluating mitigation effectiveness.

## Introduction

The study of critical transitions (tipping points) can shed light on sudden responses to slow changes in environmental drivers, which are inherent in many dynamic processes such as climate change and epidemiological outbreaks^1^. Identifying and anticipating critical transitions therefore helps to characterize a system’s propensity for unexpected shifts in its dynamics and sheds light on the longevity of the current regime^2–4^. Building such an understanding is invaluable in planning for and mitigating transitions of ever-evolving complex systems, which include regional phenomena that involve multiple interconnected social, economic, ecological, or climate drivers, where dynamical mechanisms are not completely understood^5,6^.

One such regional phenomenon is the coffee rust disease, caused by the fungus *Hemileia vastatrix*, which has periodically devastated coffee production across the globe^7^. The latest outbreak of the disease initiated in 2012–2013 and has affected coffee production throughout Mesoamerica, costing hundreds of millions of dollars to production and community livelihoods^8,9^. An examination of the ecological principles underlying the dynamics of the disease leads theoretically to the propensity of critical transition behavior^10^, and thus to the infamous inevitability of surprise so well known in crop disease history^11^.

The dynamics of the coffee rust disease are complicated^9,12^, but at a general level, local establishment involves two stages^10^, similar to other direct transmission pathogens. First, windborne urediniospores, blown from a broad region onto a farm, enter and germinate in the stomata on the underside of coffee leaves, forming haustoria that extract materials from leaf tissue, and finally exiting again through the stomata to form more urediniospores. Second, the urediniospores are dispersed to neighboring coffee bushes either through direct contact, water splash, or local turbulent wind conditions. The urediniospores may also ascend to the atmosphere above the farm and initiate the first stage in a broader spatial context.

This basic two-stage process suggests that the disease may undergo a critical transition, not only in the initial outbreak^10^, but also from high intensities to near zero, the so-called “exit” from the epidemic regime^4^, seemingly without obvious trends. As in so many other incidences of critical transitions^3,6^, a method of prediction would be useful here as evidence that management interventions designed to control the disease might be working. With scant evidence that their interventions are effective, managers may otherwise consider premature abandonment even though long-term effectiveness is on the horizon, though still obscured.

**Figure 1:**
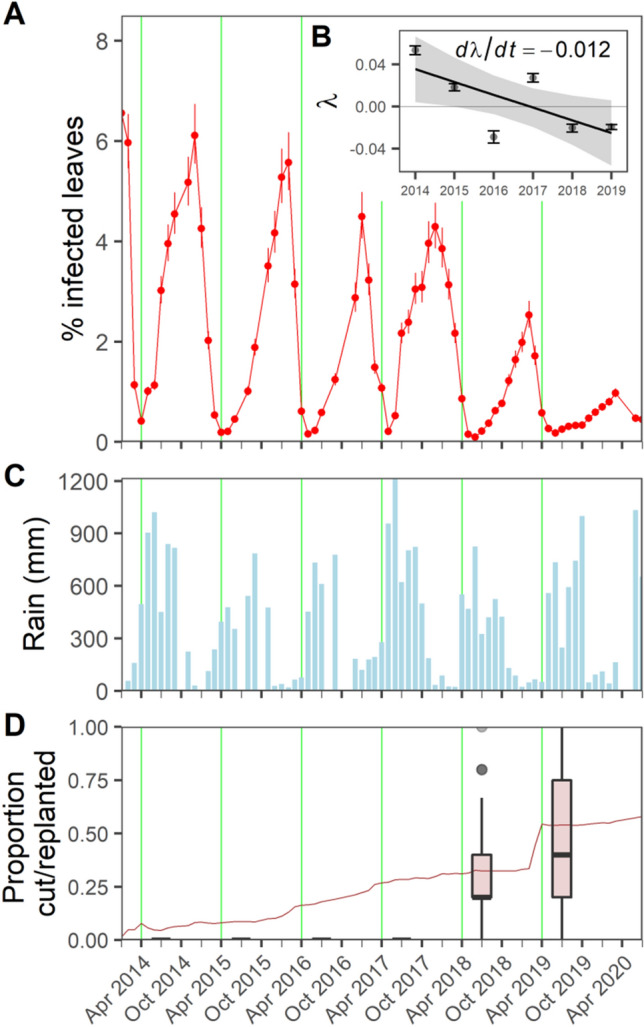
Time series of the coffee rust, monthly rainfall, resistant replanting estimates, and initial critical slowing down estimate. (**A**) Percent infected leaves (connected red points), taken from the geometric mean and standard error of 128 50 × 50 m quadrats, originally comprising 5 plants each (*n* = 640). Based on an initiation point in April (green vertical lines), we estimated (**B**) the slowing down parameter λ and its second derivative (slope and 95% confidence interval). Points and ranges represent the arithmetic mean and standard error of quadrat-level λ estimates (*n* = 128). Shown for the same time period are: (**C**) monthly rainfall and (**D**) proportion of the original 640 plants cut by farm management (line graph). We incorporated the replanted varieties that accompanied cutting in later years of the study, accounting for their effect as a covariate representing the proportion of each quadrat comprised of new plants (distribution shown as box plots for the two years they were included).

The history of the coffee rust disease in a 45 ha plot on a large organic farm (Finca Irlanda^13^) qualitatively illustrates the behavior of the disease as it has been reported throughout Mesoamerica from 2012 through 2020. Strongly seasonal, the relatively slow decline in peak mean intensity, hardly notable before 2019, became suddenly evident and declined to close to zero in 2020, suggesting a critical transition had occurred (Fig. 1A). Indeed, basic epidemiological theory^10,14^ suggests that, under the influence of forces of secular change, a critical transition might occur, not only in the outset of the epidemic, but also in its termination.

If the underlying dynamics include a bifurcation and thus a critical transition, we expect the second derivative of the ascending limb of the time series to be negative^15^. This measure is an estimation of “critical slowing down” that may precede a critical transition^3,6,15^, which is normally thought of as applied to the return of a system to quasi-equilibrium after perturbation. In the case of a seasonally forced system such as with the coffee rust, the initiation of the rust season each year can be taken as the point of perturbation, and the ascent to the peak of the year as the return of the perturbation to equilibrium. Based on this conceptualization, if the dynamics of the system are such that the approach to the equilibrium disease intensity each season is strictly secular (i.e., with no formal critical transition from bifurcation), the second derivative should be zero; whereas it will be negative in the case of critical slowing down^3^. Following Lim and Epureanu^15^ we measured the rate of slowing down as

1 $$\lambda = { }\frac{{\left( {\frac{dln\left( r \right)}{{dt}}} \right)_{ + } - \left( {\frac{dln\left( r \right)}{{dt}}} \right)_{ - } { }}}{{2{\Delta }t}}$$

where $\left( {\frac{dln\left( r \right)}{{dt}}} \right)_{ + }$ is the derivative measured before the middle of the upward trajectory, and $\left( {\frac{dln\left( r \right)}{{dt}}} \right)_{ - }$ is the derivative measured after the middle of the upward trajectory (the downward part of each oscillation is ignored). Using this framework, we examined the changes in the annual seasonal rust increase for signs of critical slowing down.

While direct application of critical transition theory may be useful in evaluating ongoing interventions, general background conditions, some beyond the manager’s control, may also contribute to secular changes in a particular parameter that would induce a critical transition. It is well known that the coffee rust is influenced by a variety of complex physical and biological factors^9,16,17^, and the dependence of spore germination on moisture is clearly one of those forces^18^. Since spore germination is known to require a critical level of moisture, an evident hypothesis is that secular changes in rainfall could be a natural force for generating a critical transition in the coffee leaf rust. It is also worth noting that, according to local informants, replanting with resistant varieties has been a general tendency in farms of the region where our study was conducted. We directly observed replanting in replacements among the sentinel coffee bushes we used to measure rust intensity. Therefore, another plausible hypothesis explaining the dynamics leading to the critical transition could also be the gradual build-up of resistant varieties of coffee. We examined associations between these two exogenous factors, rainfall (Fig. 1C) and coffee replanting (Fig. 1D), with signals of critical slowing down.

## Results

**Figure 2:**
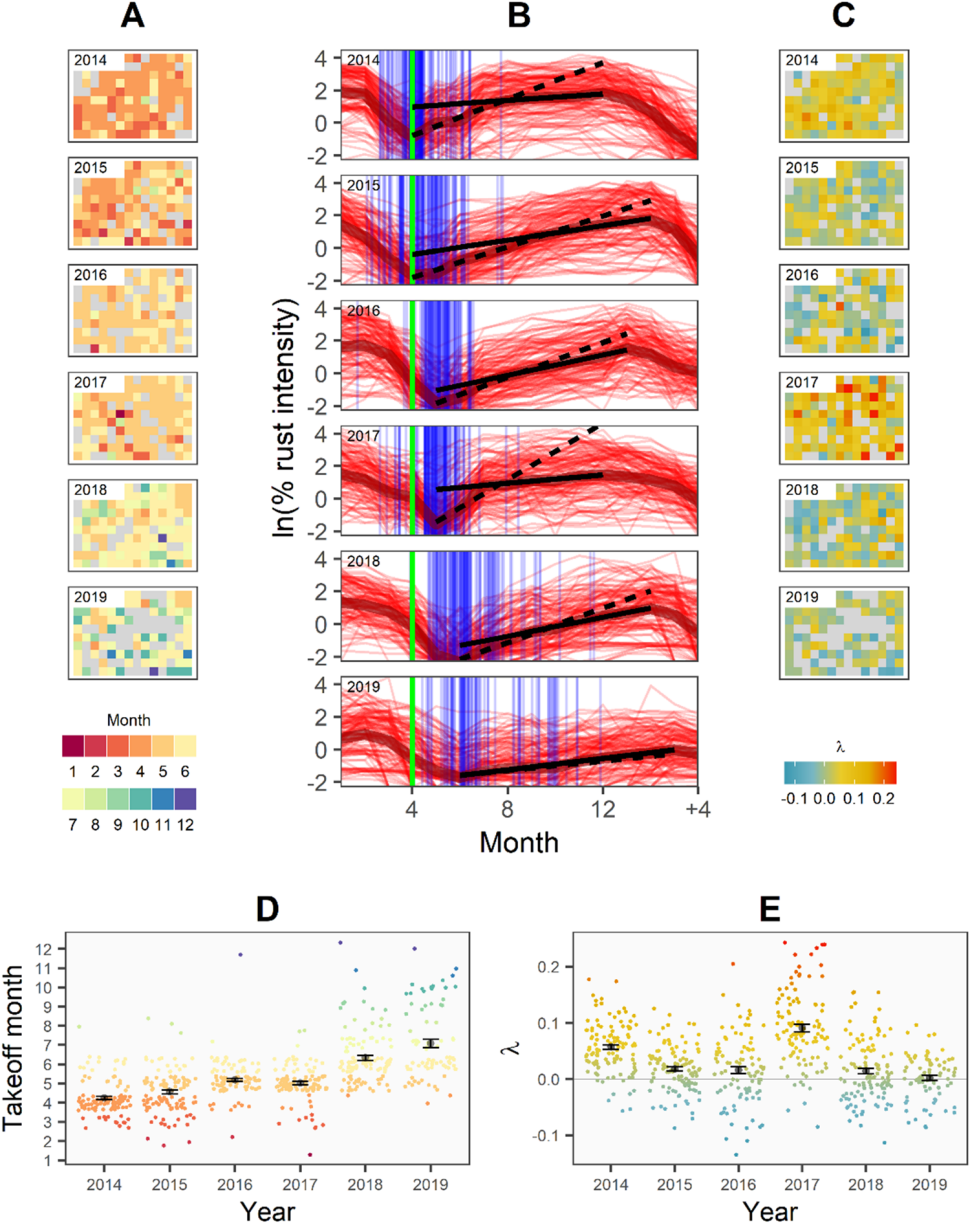
Spatiotemporal trends of critical slowing down (takeoff month and λ) from 2014–2019. Spatial plots (**A**, **C**) show the 128 50 m × 50 m sampling quadrat estimates of takeoff month (**A**) and λ (**C**) for each year (gray cells represent no trend or missing data). Time series plots (**B**) illustrate annual rust trajectories in each quadrat (thin red lines) and plot mean trends (thick red line). Heavy vertical line (green) denotes April (assumed takeoff in our initial λ estimates); blue vertical lines mark empirical quadrat-level takeoffs, jittered to convey data density. Solid and dashed slopes show derivative estimates for the first and second half of the rust season (plot mean). Their difference, normalized by the rust increase period, is equal to λ (Eq. 1), which we estimated for each quadrat (**C**). Plots (**D**) and (**E**) show the quadrat takeoff and λ values, respectively, over time (year). Color-coded points represent individual quadrat values, jittered to convey density, and black points and error bars represent the year mean and standard error.

Estimating λ for each of the six ascending limbs in Fig. 1A in a multilevel model accounting for replication at the spatial (quadrat) and temporal (year) levels, we obtained a slope of λ = − 0.012 (t = − 2.3, *p* = 0.06) for the overall plot, the basic data for which are displayed in the inset figure, Fig. 1B (further model results in Table S1). We thus found that the change in λ from 2014 to 2020 was negative, suggesting that the final decline in the 2019–2020 season is indeed a critical transition. It is notable, however, that two rates other than 2019 are less than zero, which does not conform to expectations when assuming the ascending limb is approaching its equilibrium (i.e., there is an unexpected acceleration upon approaching the peak value). This idiosyncrasy is likely due to the fixed date taken for the initiation of the increasing limb. We defined the beginning of the upward trajectory each year in our initial analysis as April, reflecting the beginning of the periods of rust increase in 2014 and 2015 and corresponding generally with seasonal patterns of rainfall increase (Fig. 1C). However, examining the relative data for the first sign of an increase in the rust each year for the 128 sampling quadrats (Fig. 2A), we found that the so-called takeoff point increased over the six-year period (Fig. 2B, D). Upon recomputing λ for each quadrat using the annual plot-level minimum (each “valley” of the disease incidence in Fig. 1A) to define the takeoff month of the rust season, we find that λ trends towards decreasing over time, with the exception of 2017 (Fig. 2C, E).

We then jointly modeled λ, corrected for the lag in disease initiation each year, and the quadrat’s actual takeoff month with covariates representing hypothesized exogenous forces: mean monthly rainfall, proportion of replanted sentinel plants, and time (years) in a multivariate Bayesian regression. From the outset of our experiment, the farm steadily cut and replaced plants most impacted by rust with rust-resistant varieties. If we take the frequency of monthly culls within our subset of sentinel plants as representative of the rate of replanting by the farm management, the yearly rate of replanting was closely linear with time (R^2^ = 0.96, Fig. S1), though with some deviation month to month (Fig. 1D). Beginning in July 2018, we began to monitor replanted, resistant plants to replace the sentinel plants that were cut. The proportion replanted covariate in our model thus accounts for the replanted individuals at the local (quadrat) spatial scale, while the overall replanting rate across the entire farm was closely related to the year variable. The distributions of the proportion replanted among our sentinel plants per spatial quadrat in 2018 and 2019 are shown in Fig. 1D.

**Figure 3:**
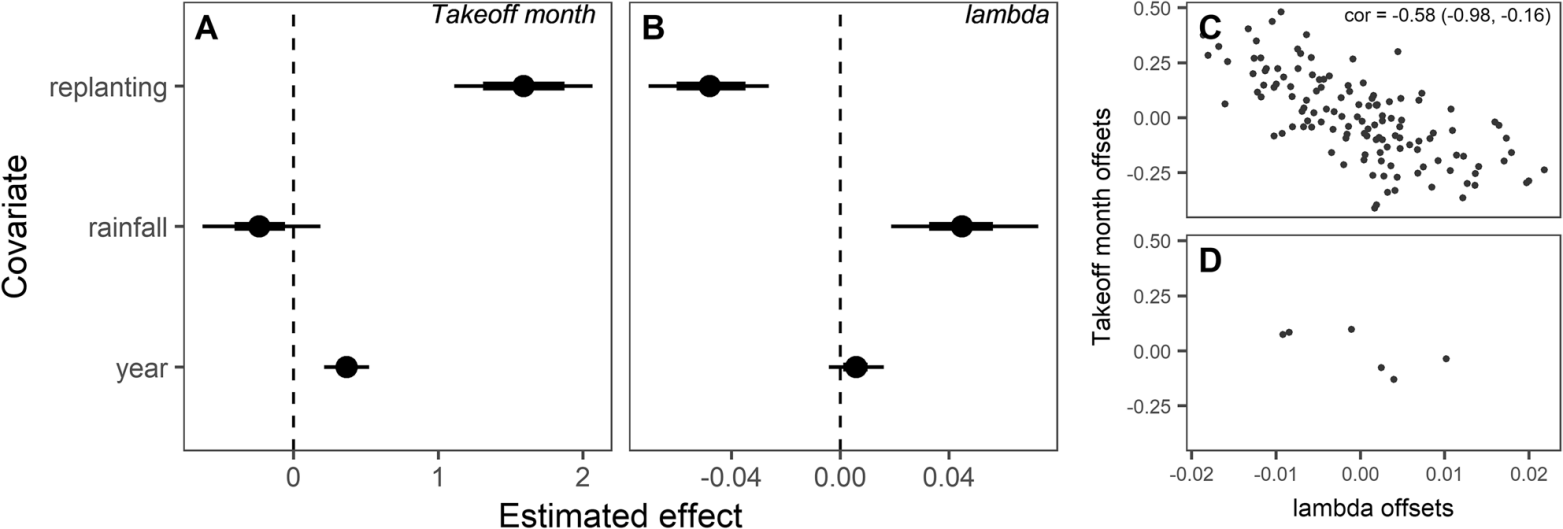
Results of bivariate multiple regression model of quadrat takeoff month and λ. Posterior estimates of covariate effects on takeoff month (**A**) and lambda (**B**), showing 66% (thick bars) and 90% (thin bars) of the highest density interval around the median (points). Covariates were proportion of sentinel plants replaced (“replanting”), mean monthly rainfall (“rainfall”, unit = 100 mm), and time (“year”, i.e., from 2013). (**C**) The model estimated group-level random effects (intercept offsets) by quadrat for the two dependent variables (median and 90% highest density interval). Correlation between year offset for the two variables (**D**) was not estimated in the final model.

When controlling for the effect of the covariates we observe a delaying effect associated with more replanted rust-resistant coffee plants as well as an increasing trend in the rust initiation over the study period (Figs. 2D, 3A). On the other hand, there is a positive relationship between λ and the annual mean monthly rainfall and a negative relationship with replanting (Fig. 3B). Accounting for these exogenous covariates, we found no significant trend in λ over time, unlike our results for the uncorrected λ. We found a strong negative correlation in the quadrat random effects between the λ and takeoff month components of the bivariate model (Fig. 3C), while the correlation between year random effect offsets was not different from zero (Fig. 3D), indicating that the slowing down signal and the slower takeoff are correlated spatially, rather than temporally. We removed the latter as an estimated model parameter because comparison between the preliminary and reduced model suggested no difference in fit (leave-one-out information criterion with parameter: − 24.7; without: − 24.8) while convergence indicators were improved (see Supplementary Online Data for model data).

## Discussion

Critical transition theory tells us that, as exogenous parameters drive the system towards a bifurcation and the emergence of a new equilibrium, we can see evidence of the upcoming transition through decreasing resistance to each oscillation peak^15^, like a ball rolling around in a cup whose walls are becoming less and less steep^1,6^. We find evidence of this critical slowing down within the year-to-year deceleration of rust growth rates just prior to their annual peak, or λ, our formal slowing down measure. This is coupled with a delay in the initiation month (takeoff) of the oscillation itself. The oscillations collapse in 2019 as the system apparently switches to a more benign non-epidemic state.

In the mid-elevation zone of southwestern Mexico where we conducted our study, the general expectation from climate change, in addition to increased temperature, is reduced rainfall^19,20^, a pattern broadly in evidence for the past five years (Fig. S2). With regard to the coffee rust disease, this precipitation trend is most closely associated with the change in λ, and reflects the obligate range of associated moisture and temperature conditions required for the rust to flourish^9,17^. On the other hand, rust-resistant replanting has been occurring throughout coffee farms in much of Mesoamerica since the rust outbreak in 2012–2013^21,22^. This could limit plant-to-plant spread by reducing opportunities for direct contact with an infected plant, as well as decrease the environmental spore load by reducing the contributing pool of infected plants in the broader region. On our site, the slowing down patterns of both λ and the rust takeoff point were associated with more rust-resistant replanting on a local level, while the significant linear delay in the year-to-year rust takeoff over the study period may reflect larger-scale effects emerging from increased resistance across the farm.

Our preliminary treatment of April as the annual rust season initiation point corresponded generally with a seasonal pattern of increasing rainfall. This approach might be assumed by a manager experiencing the system in real time without knowledge of how the rust season will play out that year. Such an assumption works well in the first two seasons of our data, where the rust increase and rainy season both appear to begin in April. However, this association decouples in subsequent years. Evidently, the critical slowing down we initially estimated in Fig. 1B is partitioned into two components: one that imposes a lag on the initiation of the disease, and another that imposes a decelerating approach to the peak rust intensity, necessitating a joint model of the initiation month and the critical slowing down warning signal (λ). That the takeoff had a significant delaying trend over the study period suggests that the initiation of the rust season can also be seen as a measure of critical slowing down, in that a slower approach to the equilibrium point (the seasonal rust peak) will likely be reflected in a failure to even recognize the increase when it is still very small. Thus, the conspicuous change in takeoff point over time (Fig. 2A, B) itself suggests a critical slowing down and the negative values of λ in Fig. 1B may be mainly a reflection of a lag in takeoff time. The overall increasing lag in rust takeoff each year could mean that the transmission factor itself may be exhibiting a critical slowing down as the critical transition is approached, due to the progressive influence of parameters outside of the present analysis. Given the basic biology of the disease^10^, this trend may stem from a year-to-year secular decline in the environmental spore load causing initiation of the disease season to be deterred by a small amount each year.

**Figure 4:**
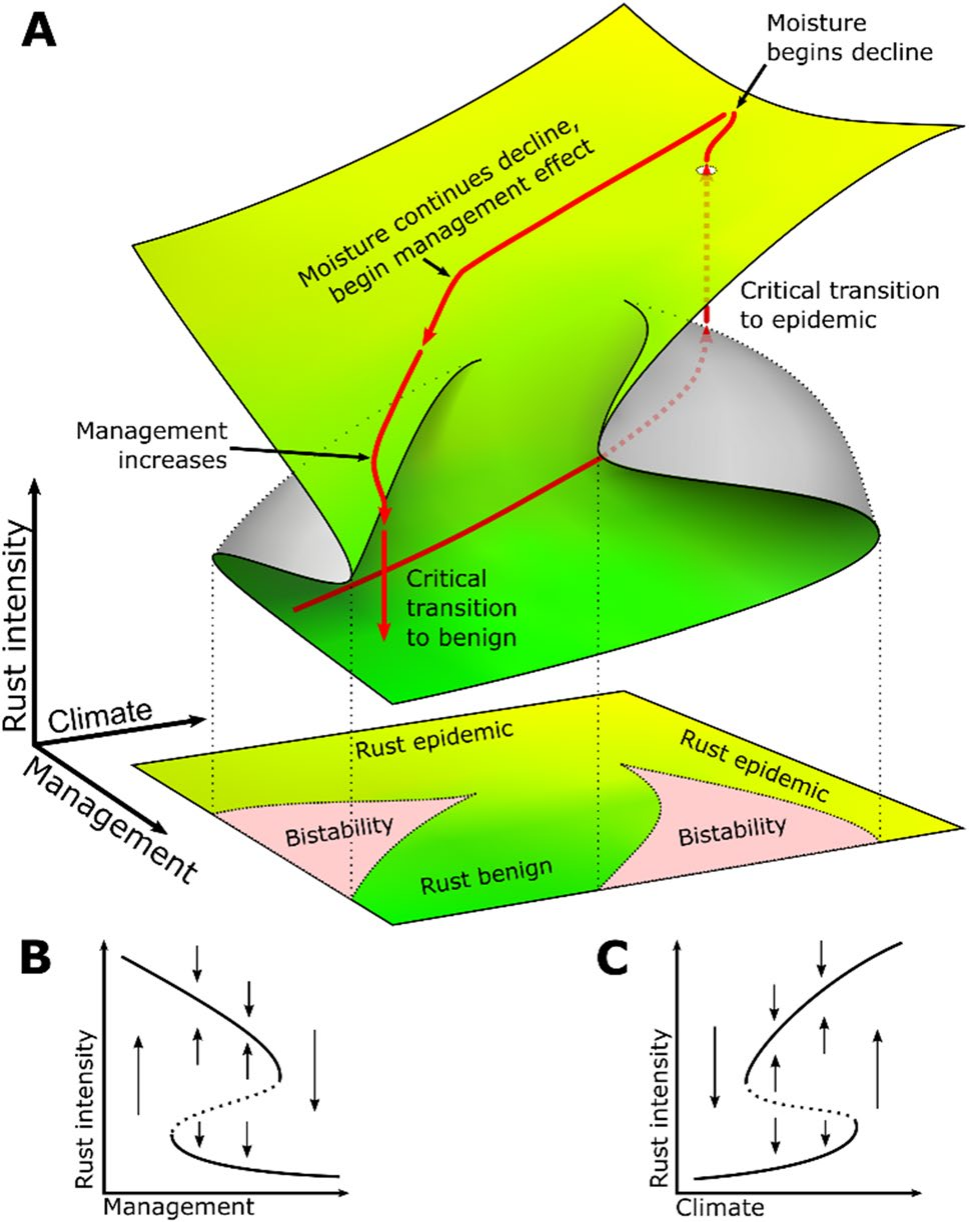
Envisioning the combination of climate and management effects in a joint hysteretic framing, stemming from gradual change in both forcing parameters. (**A**) Relative positions of rust intensity for each year are illustrated in their approximate positions with red arrows (other trajectories could be imagined based on the data presented herein). Inset plots provide a conceptualized view of the dynamics between the two forcing variables on rust intensity: (**B**) management (in our case, resistant variety replanting) and (**C**) climate (precipitation). In the inset plots, arrows indicate directions of change; solid and dashed lines indicate stable and unstable equilibria, respectively.

The evident relationship between critical slowing down and reduced rainfall is suggestive of a connection to climate change, while a slow secular increase in the proportion of rust-resistant varieties could have inhibited local or regional spread, leading to an effective “herd immunity” to the disease. The joint operation of reduced rainfall and fewer susceptible plants raises the possibility of dual bifurcations and hysteretic zones in the dynamic landscape, which we illustrate in a qualitative fashion (generalized to climate and management) in Fig. 4a. The initial outbreak, which was unexpected in light of the historically low, but persistent, levels of rust in the region^7,8^, was likely a critical transition precipitated by interactions between local and regional processes^14^. As foregrounded in Fig. 4b, we propose that a possible parameter driving the system to this initial bifurcation was recent increases in precipitation, as evidenced in local rainfall records (Fig. S2) and regional trends^19^. This could have propelled the system past a hysteretic phase space to where seasonal conditions dictated that the system jump to a high rust intensity equilibrium, represented conceptually in Fig. 4a by the dotted trajectory leading to the upper surface of the landscape that corresponds to an epidemic state of the rust. Likewise, in the years following the outbreak, our findings suggest that, while the system still tracked precipitation, progressive replanting of resistant varieties emerged as another parameter axis (management) that drove the system through a second critical transition back to a low rust equilibrium (Fig. 4c). Although the dynamical landscape in Fig. 4 is a qualitative representation, the trajectory along the upper surface helps to visualize how two exogenous forces, operating separately, both contributed to the critical transition we observed in our data. The trajectory we propose brings attention to the interesting possibility that the main driver of slowing down shifted from climate (precipitation) to management (replanting), leading to the second critical transition. Indeed, though the average yearly replanting rate remained roughly the same year-to-year (Fig. S1), we note that much of the cutting prior to the collapse in 2019 seemed to be concentrated in April that year (Fig. 1D), accompanied presumably by a similarly timed replanting campaign.

Recent work on critical transitions suggests that perturbances driving a system to transition are more realistically not distinct or isolated, and that the stochastic and deterministic elements of the system can therefore be entangled or even interdependent^4^. Likewise, we find that the variability in the environmental covariates of monthly rainfall and resistant variety replanting better explained patterns in λ than a linear trend leading up to the transition, as represented by the year variable. The correlation between the quadrat grouping offset estimates from the λ and takeoff components of the multivariate model also suggest that slowing down and delayed takeoff were associated at the individual quadrat level (Fig. 3C). Accounting for this spatial effect, these two components do not appear to be correlated by year (Fig. 3D). This suggests that the shared variability between these two indicators reflects variability in spatial environment within the plot rather than idiosyncratic effects of unique years. Besides the direct effect of resistant varieties, local stochasticity and spread dynamics may also play a role. Local growing conditions, such as variability in shade from overstory trees, can affect dispersal through rainfall splash and wind^23,24^. Additional management factors may also play a role, such as the vegetation structure and the presence of paths^25^, as well as the physical relationship between coffee plants^26^.

Our observations of the rust dynamics themselves allow us to detect the general signals anticipating a critical transition, though the drivers may emerge from a complex system of dialectical interactions that must be considered in their whole^7,27^. The concept of critical slowing down thus may lend itself to application across coffee-growing regions, where predicted effects of climate change and other geographic conditions may differ^9,19^. Since the emergence of the rust outbreak, recommendations and protocols have been published for monitoring rust levels, potentially providing managers with regular data in changes in rust intensity for many areas^9,28^. As the resilience of a system can be interpreted through measuring critical slowing down prior to catastrophe^2^, as well as, in our case, the “exit time” from an undesirable regime^4^, we demonstrate that such concepts may be applied to this monitoring data to gain some insight into the system’s status. Future studies could explore signs of critical slowing down across coffee-growing regions and management systems to see how these signals predict significant changes and respond to local drivers, potentially adding to the vocabulary of agroecological management.

In sum, it is clear that both a lag in takeoff point for the seasonal oscillation and the rate of approach to the peak each year seem to conspire to produce a critical slowing down, strong evidence that the decline in the disease in 2019–2020 is indeed a critical transition, regardless of the underlying mechanism. While our model suggests that two exogenous forces, rainfall and resistant variety replanting, may be driving the slowing down in our case, the underlying dynamical landscape is likely not unique to our site. More generally, the phenomenon of multivariate bifurcations leading to subsequent critical transitions (e.g., Fig. 4) is perhaps more common than thought^29^. Examinations of critical transitions should therefore consider the larger dynamical landscape for the possibility of subsequent transitions.

## Methods

### System and dataset

In a 45-ha plot previously established for another purpose, we identified a central shade tree in each of 128 “quadrat” 50 m × 50 m subplots (eliminating the border plots). Around that shade tree we selected five sentinel coffee bushes to monitor monthly. Each month the total number of leaves on the bush was estimated visually and the number of leaves with one or more lesions of the coffee rust was tabulated nondestructively. The farm is a certified organic farm (Finca Irlanda) growing *Coffea arabica*. The composition of *C. arabica* varieties on the farm is heterogeneous, reflecting historical management decisions and replantings over time. Therefore, the genetic lines of our sentinel plants were not known exactly, but based on informal field observations, likely included a mixture of Typica, Bourbon, Caturra, and Catuai. Resistant varieties found on the farm included Catimor, but other resistant varieties may have also been present. We account for possible variability arising from different compositions of genetic lines in each quadrat in our statistical analyses by including an intercept offset random effect for each quadrat.

In response to the coffee leaf rust outbreak in 2012/2013, the farm began to cut down diseased and susceptible individuals and replacing them with resistant varieties. Although cutting occurred continuously from the beginning of the study, we did not originally replace the culled individuals of our sentinel plants because we were tracking the same individuals in each quadrat over time. However, once the proportion of our sentinel plants that were cut exceeded 25% (approximately 1.4 plants per quadrat in June 2018), we began to incorporate replanted individuals into our dataset, starting in July 2018. We calculated the proportion of new individuals in a given quadrat (normalized by the local extant plants) in the beginning of the rust season each year, which we used as a covariate in the final model. We also calculated monthly rainfall aggregated from a weather station on the farm, which we summarized as a yearly average. All monitoring complied with relevant institutional, national, and international guidelines and legislation for research conducted on plants.

The farm applies calcium carbonate laced with copper sulfate, as is common in organic farms in the area, to generally combat fungi and, since 2012, was specifically aimed at the coffee rust fungus. These applications occurred between March and May each year, though we do not have access to the exact dates. Although the timing of the application may have had an influence on each year’s rust increase, it is unlikely that it would have been with the regular advancement that we observed in the gradual increase in rust takeoff each year. Any other year-to-year pattern in fungicide application would then be attributed to the year random effect in our models.

### Statistical analysis

We conducted analyses on monthly rust intensity (% of total infected leaves) summarized to the quadrat level from the geometric means of the five sentinel plants in each of the 128 quadrats from 2014 to 2020. Our initial model assumed that the seasonal rise in rust intensity began in April each year, which is reflected in trends for 2014 and 2015 and generally coincides with the initiation of the rainy season. Based on plot-wide means (Fig. 1), we identified the “peak” intensity month of each year. We then fit an estimate of λ according to Eq. (1) for the period between each April and the subsequent annual peak in each quadrat using maximum likelihood linear regression in the statistical software R^30^. To do this, we subset the months from April to the peak into first and second halves. For each quadrat and year, we fit the equation,

2 $$\ln \left( {\text{r}} \right) = \beta_{0} + \beta_{1} t_{i} + \beta_{2} t_{i} {*}half_{i} + \epsilon$$

where ln(r) was the natural log of the % rust intensity, *t*_*i*_ was the time in period *i*, *half*_*i*_ was an indicator variable that took the value 1 for data in the first half and 0 otherwise, and ε was the normally distributed error. For seasons with an odd number of months, we included the median month in both subset halves. We did not estimate λ for quadrat/year combinations with less than 5 months of available data (n = 6), i.e. with less than three data points per subset. We calculated λ by dividing the estimated coefficient *β*_*2*_ in Eq. (2) by the total period covered by the two halves (the entire increasing rust period), i.e., 2Δt in Eq. (1). In turn, we used the modeled λ values as the dependent variable (n = 762) in a linear mixed effect model to estimate the second derivative of λ by fitting a coefficient for year as a continuous fixed effect. This model included group-level random effect intercept offsets to account for correlated error at the individual quadrat (n = 128) and year levels (n = 6). This mixed effect model was fit with the R package “lme4”^31^. Further model results can be found in Supplementary Information Table S1.

We re-computed λ and accounted for the shifting takeoff time by adjusting the window of data included in Eq. (2) based on plot-wide mean trends. We also systematically identified the unique takeoff months for each quadrat and year by applying a moving window filter to the time series data for each quadrat. The filter identified takeoff points in the time series as months that were not followed by lower values in the next five months and were preceded by five months of equal or greater rust levels, i.e., the lowest month preceding a general increase within an approximately year (11-month) window. We did not consider cases with missing data within the window that exceeded 40% and excluded one case where rust increase was preceded by several months of missing data.

We modeled quadrat takeoff month and the adjusted quadrat λ as dependent variables (n = 610) in a Bayesian bivariate linear regression with the R package “brms”^32^, which uses a Hamiltonian Monte Carlo algorithm implemented by the package “RStan”^33^. The model estimated the fixed covariate effects of a continuous variable for year, the proportion of replanted bushes in a quadrat, and the mean monthly rainfall. This model also fit group-level intercept offsets for individual quadrats and years that were allowed to correlate between the two dependent variables. We used default weakly informative diffuse priors^32^ and sampled from four Monte Carlo Markov chains, each with 6000 iterations including 3000 warmup iterations. Posterior distribution estimates were checked for convergence and adherence to model assumptions. Further model results are provided in Supplementary Information Table S2.

## Supplementary Information

Supplementary Information.

## Data Availability

Analysis code, model data, and associated data are available at https://figshare.com/s/97fe2c5495e11e77c74e.
