# Supplementary material for: Reduced rainfall and resistant varieties mediate a critical transition in the coffee rust disease
Source: Sci Rep. 2022 Jan 28;12:1564. doi: 10.1038/s41598-022-05362-0 (PMC8799695; doi:10.1038/s41598-022-05362-0)
Supplement: Supplementary file 1 — Supplementary Information. [file 41598_2022_5362_MOESM1_ESM.docx]

**Supplementary Materials for:**

Reduced rainfall and resistant varieties mediate a critical transition in the coffee rust disease

**Authors:** Kevin Li*, Zachary Hajian-Forooshani, Chenyang Su, Ivette Perfecto, and John Vandermeer

**Corresponding author:** Kevin Li, [likevin@umich.edu](mailto:likevin@umich.edu)

**This file includes:**

Fig. S1

Fig. S2

Tables S1 to S2

SI References


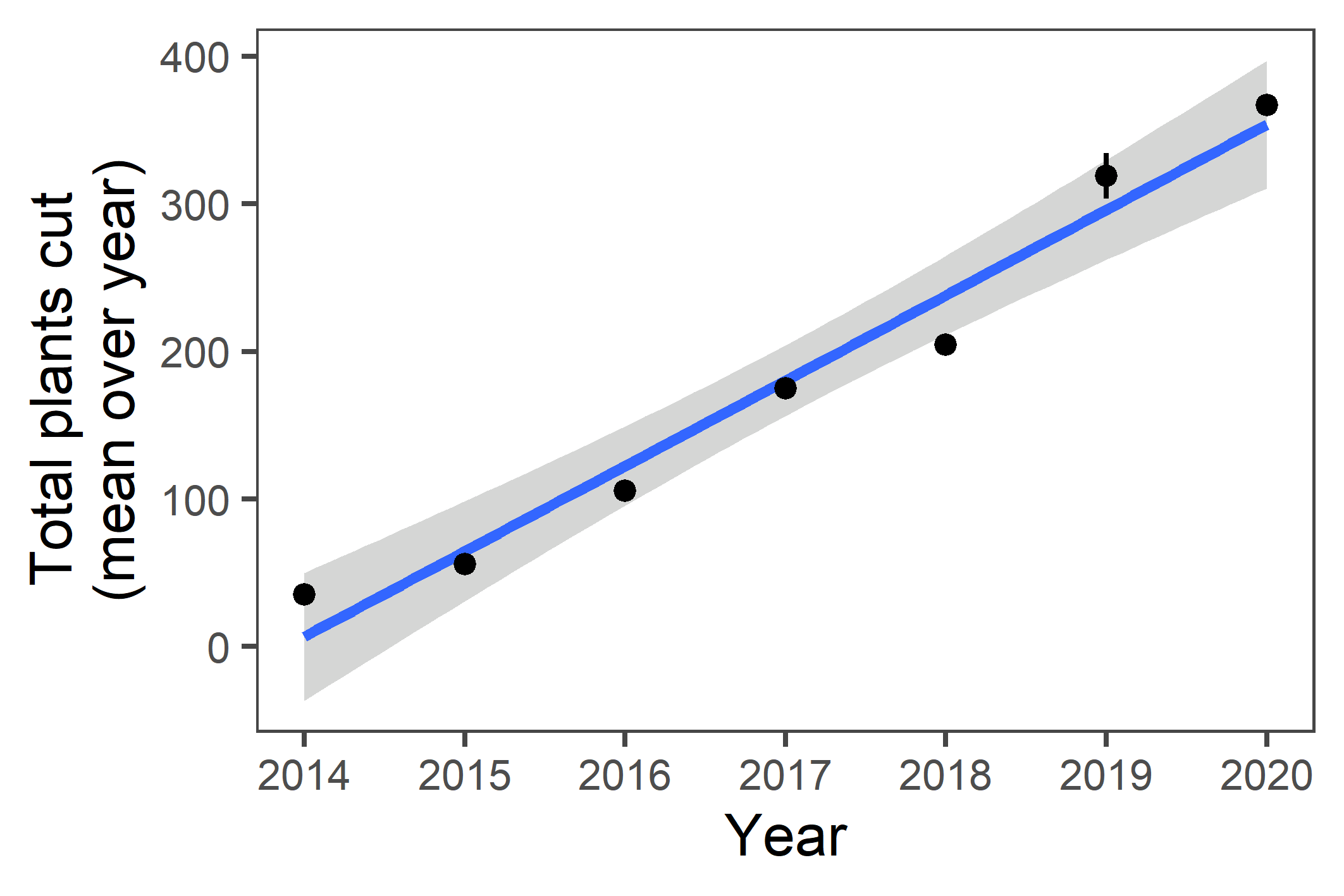


**Fig. S1.** Trend in total number of plants cut (yearly mean) within our sentinel plant dataset. Year-to-year increase in cut plants followed a strongly linear trend (57.8 plants/yr, *F* = 153.7 (1, 5), *p* < 0.001, R^2^ = 0.96). Since cutting was accompanied by replanting with rust-resistant coffee varieties, this trend suggests that the increase in resistant plants within the farm was also strongly linear.


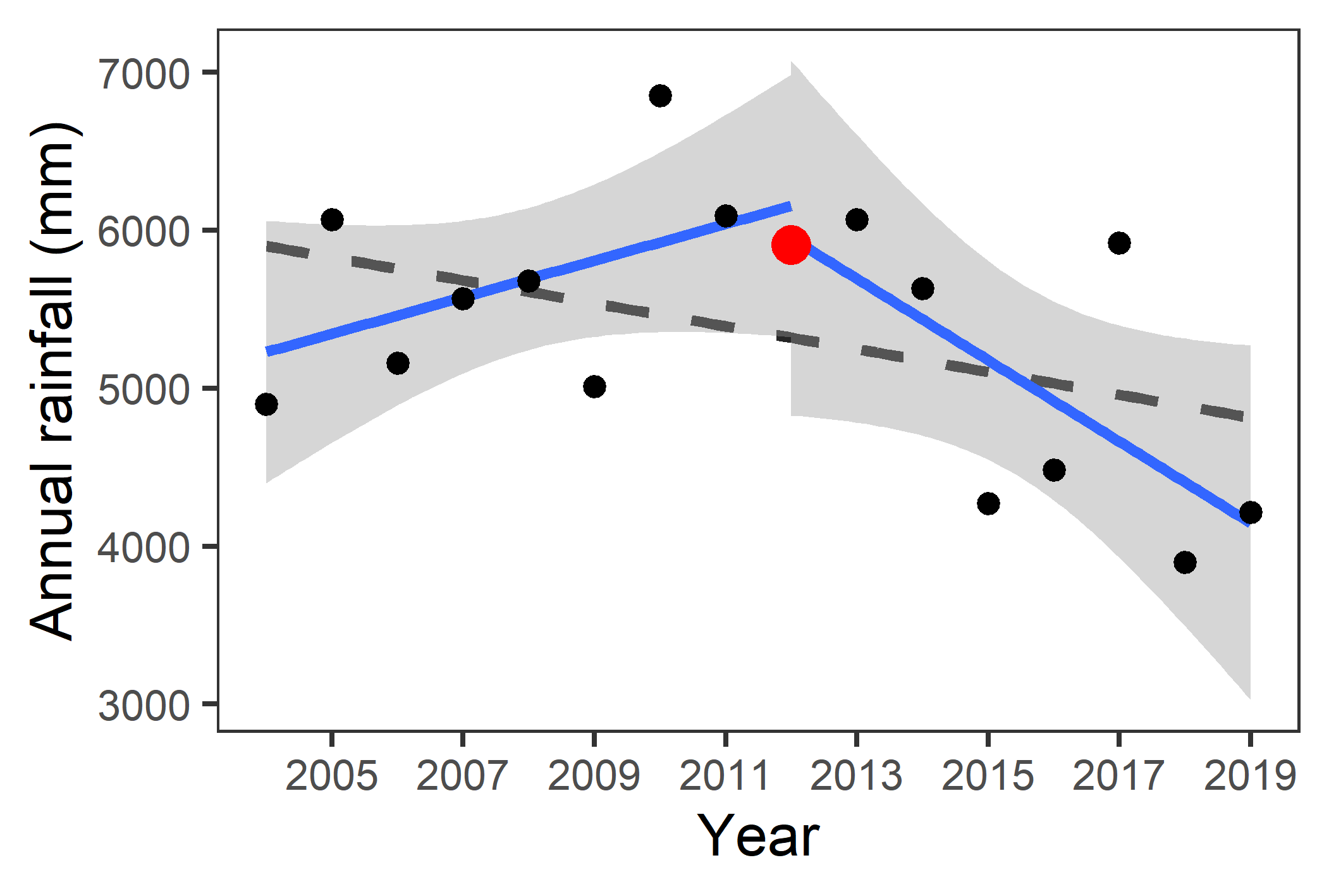


**Fig. S2.** Small-scale annual rain trends (indicated by blue lines with 95% confidence region) before (2004-2012) and after (2012-2019) the rust outbreak in 2012 (large red point). Leading up to rust outbreak, annual rain had a rising trend (116 cm/yr, *F* = 2.46(1, 7), *p* = 0.16, R^2^ = 0.15). Proceeding from the outbreak, rain had a marginally significant decreasing trend (-258 cm/yr, *F* = 5.51(1, 6), *p* = 0.06, R^2^ = 0.39). Over the entire period of 2004-2019 (dotted grey line), annual rainfall tended to decrease (-72 cm/yr, *F* = 2.89(1, 14), *p* = 0.11, R^2^ = 0.11).

**Table S1.** Linear mixed effect model results for initial model assuming rust season start in April of each year. The response variable was the estimated λ value for each quadrat (n=762). Some quadrats in some years were dropped due to missing data (see Methods). Random effects included a random intercept offsets for year (n = 6) and quadrat (n = 128). The model had a marginal R^2^ value of 0.15 (proportion explained variance attributed to fixed effects only) and a conditional R^2^ of 0.38 (proportion explained variance of all effects)^1^.

|  | Estimate | Std. Error | df | t-value | Pr(>\|t\|) |
| --- | --- | --- | --- | --- | --- |
| **Fixed effects** |  |  |  |  |  |
| Intercept | 0.047 | 0.020 | 6.047 | 2.328 | 0.059 |
| Year | -0.012 | 0.005 | 5.996 | -2.321 | 0.059 |
|  |  |  |  |  |  |
| **Random effects (group intercepts)** | | |  | | |
|  | sd |  |  |  |  |
| Quadrat | 0.015 |  |  |  |  |
| Year (factor) | 0.022 |  |  |  |  |

**Table S2.** Results of Bayesian bivariate multiple regression of quadrat takeoff month and lambda, fit to exogenous variables of year (“Year”), average monthly rainfall (“Rain”, 100mm/month), and proportion replanted coffee plants in quadrat (“Planting”). Random effects included intercept offsets for year (n = 6) and quadrat (n = 128). Correlation of quadrat-level intercept offsets between the dependent variable components was estimated as part of the model. The Bayesian-equivalent^2^ marginal R^2^ (fixed effect only) of our final model was 0.42 (95% CI: 0.32, 0.51) for takeoff month and 0.27 (95% CI: 0.11, 0.41) for lambda. With the random effects included, these R^2^ values were, respectively, 0.47 (0.42, 0.51) and 0.33 (0.28, 0.39).

|  | Estimate | Est. error | Lower 95% CI | Upper 95% CI | Rhat | Bulk_ESS | Tail_ESS |
| --- | --- | --- | --- | --- | --- | --- | --- |
| **Fixed effects: Takeoff month** | | | | | | | |
| Intercept | 4.92 | 1.46 | 2.39 | 7.74 | 1.00 | 3439 | 1549 |
| Year | 0.36 | 0.11 | 0.15 | 0.58 | 1.00 | 5356 | 2990 |
| Rain | -0.25 | 0.33 | -0.86 | 0.29 | 1.00 | 3624 | 1565 |
| Planting | 1.59 | 0.30 | 1.00 | 2.17 | 1.00 | 10714 | 9246 |
|  |  |  |  |  |  |  |  |
| **Fixed effects: Lambda** | | | | | | | |
| Intercept | -0.16 | 0.10 | -0.35 | 0.01 | 1.00 | 4920 | 2780 |
| Year | 0.01 | 0.01 | -0.01 | 0.02 | 1.00 | 5296 | 2819 |
| Rain | 0.05 | 0.02 | 0.01 | 0.09 | 1.00 | 5199 | 3008 |
| Planting | -0.05 | 0.01 | -0.07 | -0.02 | 1.00 | 12132 | 9859 |
|  |  |  |  |  |  |  |  |
| **Random effects: Quadrat (group intercept)** | | | | | | | |
| sd(Takeoff month) | 0.34 | 0.09 | 0.12 | 0.51 | 1.00 | 2099 | 2123 |
| sd(lambda) | 0.02 | 0.00 | 0.01 | 0.02 | 1.00 | 2384 | 2008 |
| correlation | -0.53 | 0.29 | -0.95 | 0.19 | 1.00 | 1896 | 2183 |
|  |  |  |  |  |  |  |  |
| **Random effect: Year (group intercept)** | | | | | | | |
| sd(Takeoff month) | 0.29 | 0.32 | 0.02 | 1.07 | 1.00 | 1941 | 1508 |
| sd(lambda) | 0.02 | 0.02 | 0.00 | 0.08 | 1.00 | 2320 | 1945 |

# SI References

1. Nakagawa, S. & Schielzeth, H. A general and simple method for obtaining R2 from generalized linear mixed-effects models. *Methods Ecol. Evol.* **4**, 133–142 (2013).

2. Gelman, A., Goodrich, B., Gabry, J. & Vehtari, A. R-squared for Bayesian Regression Models. *Am. Stat.* **73**, 307–309 (2019).
